# Burden assessment of podoconiosis in Wayu Tuka woreda, east Wollega zone, western Ethiopia: a community-based cross-sectional study

**DOI:** 10.1136/bmjopen-2016-012308

**Published:** 2016-09-23

**Authors:** Kenate Bekele, Kebede Deribe, Tsige Amberbir, Geleta Tadele, Gail Davey, Abdi Samuel

**Affiliations:** 1 Department of Microbiology, Wollega University, College of Medical and Health Sciences, Nekemte, Ethiopia; 2 Wellcome Trust Brighton & Sussex Centre for Global Health Research, Brighton & Sussex Medical School, Falmer, Brighton, UK; 3 Addis Ababa University, School of Public Health, Addis Ababa, Ethiopia; 4 International Orthodox Christian Charities (IOCC), Addis Ababa, Ethiopia; 5 Department of Parasitological, Wollega University, College of Medical and Health Sciences, Nekemte, Ethiopia

**Keywords:** Acute adenolymphangitis, Podoconiosis, elephantiasis, Ethiopia

## Abstract

**Objective:**

Podoconiosis is a neglected tropical disease characterised by a slowly progressive swelling of the foot and lower leg. It is prevalent among subsistence barefoot farmers who live and work in highland areas of the tropics. This study was conducted in Wayu Tuka ‘woreda’ (district), western Ethiopia to determine the prevalence of podoconiosis and assess factors associated with acute adenolymphangitis (ALA) episodes.

**Methods and analysis:**

A two phase, community-based cross-sectional study was conducted between January and March 2015. First, all households in the district were surveyed to determine the prevalence of podoconiosis. This was followed by a second phase in which 366 people with podoconiosis from four randomly selected ‘kebeles’ (subdistricts) were assessed for clinical features of the disease, shoe-wearing habits, personal hygiene, social stigma and functional impairment. Data entered into Epi DATA were then exported to SPSS. Logistic regression analysis was conducted to identify factors associated with ALA.

**Results:**

Prevalence of podoconiosis in the population was 3.05% (1197/39 256) (95% CI 2.9% to 3.2%). The prevalence was significantly higher among women than men (3.67% vs 2.4%). Most (92.2%) people with podoconiosis were in the economically active age group (15–64 years) in the first phase survey. Of participants in the second phase of the study, 43% had stage 2 disease and 38.1% had ‘moss’-like skin changes. On average, people with podoconiosis had 23.3 episodes of ALA/year and each person with podoconiosis lost 149.5 days of activity/year. Never walking barefoot and daily foot washing were both associated with decreased odds of ALA (AOR=0.23; 95% CI 0.06 to 0.80 and 0.09; 95% CI 0.01 to 0.75, respectively).

**Conclusions:**

A relatively high prevalence of podoconiosis, frequent ALA episodes and considerable decreases in daily activities were identified in this district. Footwear use and daily foot hygiene were associated with decreased odds of ALA. We recommend prevention and morbidity management interventions to address this developmental challenge.

**Strengths and limitations of this study**

- The study used a complete census of all podoconiosis cases in one district. It provides a profile of people with podoconiosis in the district, which is important for rational deployment of limited resources towards prevention and treatment of the disease.
- A limitation of the study is case identification—this was not supported by serological tests to exclude lymphatic filariasis.
- A second limitation was that self-report of acute lymphangioadenitis was not validated in any way.

## Background

Podoconiosis is a disabling and stigmatising neglected tropical disease (NTD) which affects the lower limb. It is caused by long-standing exposure to red clay soil of volcanic origin.^1^ It is characterised by the development of persistent swelling of the foot which progresses to the dorsum and gradually extends up the lower leg. Podoconiosis affects the lower limbs, and the swelling is usually limited to below the knees.^2^ ^3^

Podoconiosis can be distinguished from filarial elephantiasis through history and clinical examination: it develops first in the foot, it causes bilateral but asymmetric swelling often confined to the lower leg and groin involvement is rare. In contrast, the swelling of lymphatic filariasis is commonly found above the knee and often involves the groin. Another common differential diagnosis is leprosy lymphoedema, but podoconiosis can be distinguished from this because sensation persists in the toes and forefoot, and trophic ulcers, thickened nerves and hand involvement are not found.^4^

Acute adenolymphangitis (ALA) is a common and disabling complication of podoconiosis lymphoedema, yet has been very little investigated until now. According to studies conducted in northern and western Ethiopia, patients with podoconiosis experienced on average five episodes of ALA per year and up to 90 days per year incapacitated by ALA.^5^ ^6^ In filarial lymphoedema (LF), episodes of ALA have been shown to accelerate damage to peripheral lymphatic vessels and to lead to fibrosis.^7^

Prevalence of podoconiosis is high in many highland parts of Africa: Cameroon,^8^ Ethiopia.^9^ In Ethiopia, prevalence of podoconiosis is about 5% in areas with irritant soil;^1^ 11 million Ethiopians (18% of the population) are at risk through exposure to the irritant soil^1^ and up to 90% of affected individuals are from the most economically active age groups.^5^ In a study in southern Ethiopia, it was found that affected individuals lose 45% of their total productive work days. Direct and productivity costs of podoconiosis in a group of 1.5 million inhabitants have been estimated at US$16 million a year, imposing an economic burden of $208 million per year in Ethiopia.^10^

Recently, podoconiosis and other NTDs have been receiving attention in Ethiopia.^11^ The Federal Ministry of Health of Ethiopia endorsed inclusion of podoconiosis in the National Master Plan for Neglected Tropical Diseases in 2011, and nationwide mapping of podoconiosis and lymphatic filariasis was conducted in 2013.^11–13^ While the ‘woreda’-level burden of disease as manifested by the leg swelling has been described in several recent studies,^5^ ^6^ ^14^ very little attention has been given to ALA, another important sequela of podoconiosis. This study aimed to determine the burden of both podoconiosis lymphoedema and ALA in western Ethiopia.

## Methods

### Study area and period

The study was conducted in Wayu Tuka ‘woreda’ (an equivalent of a district in Ethiopia) in east Wollega Zone, Oromia Region, western Ethiopia. It is located 324 km from the capital Addis Ababa at an altitude of 1700–2200 m above sea level and has an average annual rainfall of 2400 mm. The population of Wayu Tuka ‘woreda’ is estimated at 75 970, living in 15 930 households,^15^ of whom 95% are in rural areas and depend on subsistence farming for their living. This study was conducted from January to March 2015. The study is reported according to the STROBE Statement—checklist for cross-sectional studies (see online supplementary file 1).

10.1136/bmjopen-2016-012308.supp1 supplementary file

### Study design and sampling

A two phase cross-sectional community-based study was conducted. In the first phase, all the households in the ‘woreda’ were surveyed to determine the prevalence of podoconiosis and to identify peoples with podoconiosis for the second phase study. In the second phase, 366 people with podoconiosis from four randomly selected ‘kebeles’ (an aggregate of villages and the smallest administrative unit in Ethiopia) were approached to assess clinical features of the disease, habits of shoe-wearing, personal hygiene social stigma due to the disease and functional impairment.

### Inclusion and exclusion criteria

All individuals who were willing to participate were included, however, in the second phase; people with podoconiosis with mental health problems precluding them answering questions with substantial recall were excluded.

### Data collection

Thirty level 4-holder nurses employed as health extension workers and two Bachelor of Science (BSc) nurse supervisors were recruited in the first phase survey, while 10 health extension workers and two BSc nurse supervisors were recruited for the second phase survey. The health extension workers were responsible for house-to-house enumeration of podoconiosis cases and the interviewing and physical examination of people with podoconiosis. The nurses were responsible for supervising the activities of the health extension workers. In households where there was more than one person with podoconiosis, all of them were invited to the interview and physical examination including measurement of leg circumference. All field staff were trained for 1 day before carrying out data collection. The questionnaire included sections from earlier studies on podoconiosis (see online supplementary file 2)^5^ ^6^ ^14^ including history of condition, understanding about causes, stigma, family history and preventive behaviours. The questionnaire was prepared in English, translated into Afaan Oromo (the local language) and translated back to English to maintain consistency of the variables under question. Health extension workers were trained to take informed consent from participants, to elicit features that differentiate lymphoedema and leg swelling resulting from podoconiosis from other diseases such as leprosy and lymphatic filariasis, to conduct clinical staging according to a standard method, to assess for presence of ALA and to measure leg circumference. A pretest performed in Jalallee kebele, in a neighbouring district, ensured reliability of enumeration and diagnostic skills.

10.1136/bmjopen-2016-012308.supp2 supplementary file

### Operational definitions

ALA: A reddish, hot, swollen leg with a painful groin.^5^ ^16^

Chagino: The time around the new moon, often associated in local understanding with weather changes.^5^

Economically active age: Anyone between the ages of 15 and 65 years.^10^

Leg circumference: The largest circumference between the level of the ankle and the knee measured using a tape, to a precision level of the nearest centimetre.^17^

Mossy lesions: Papillomatous horny lesions giving the skin a rough appearance.

A person with podoconiosis: An individual in an endemic area diagnosed by a trained nurse and a health extension worker who fulfils all of the following diagnostic criteria: history of burning sensation in the feet when the swelling started; visible swelling that started at the feet and progressed upwards; with at least stage one of the five clinical stages of podoconiosis; and with no known clinical signs or symptoms of leprosy or lymphatic filariasis.^9^

### Data processing and analysis

Data were entered into Epi DATA, then exported to SPSS data V.20 and summarised in tables. To identify risk factors associated with ALA, a bivariate logistic regression was performed, and then multivariable logistic regression was conducted. A significance level of 0.05 was used as a cut-off.

### Ethical considerations

After discussing the study with the local authorities, we obtained written approval to conduct the study in all ‘kebeles’. The ultimate purpose of the study was explained and data were collected only from those who gave written consent. The participants were informed of their rights including ability to stop participating at any time or to skip questions they do not wish to respond to. For individuals <18 years of age, consent was obtained from parents or guardians. People with podoconiosis were advised to attend health clinics for management of the condition.

## Results

### Sociodemographic characteristics of people with podoconiosis in the phase 1 survey

The male to female ratio was 1:1.58. The mean (±SD) age of people with podoconiosis was 43.1±13.3 years. Slightly more than 9 in 10 (1117, 93.3%) belonged to the economically active age group. The majority (95%) lived in rural areas and were reliant on subsistence farming (table 1).

**Table 1:** Sociodemographic characteristics of people with podoconiosis in the phase 1 survey in Wayu Tuka ‘woreda’, January to March 2015 (N=1197)

Characteristics	Category	N (%)
Marital status	Single	131 (10.9%)
	Married	905 (75.6)
	Divorced	25 (2.1)
	Widowed	136 (11.4)
Family size	1–4	653 (53.9)
	≥5	559 (46.1)

### Burden of podoconiosis

Prevalence of podoconiosis in the population was 3.05% (1197/39, 256, 95% CI 2.9% to 3.2%). The prevalence was significantly higher among women than men (3.67% vs2.40%). Prevalence disaggregated by age and sex is shown in table 2.

**Table 2:** Prevalence of podoconiosis disaggregated by age and sex in Wayu Tuka woreda

	Male			Female		
Age	Number examined	People with podoconiosis	Prevalence (%)	Number examined	People with podoconiosis	Prevalence (%)
15–24	8025	10	0.1	8801	9	0.1
25–34	4574	33	0.7	4516	83	1.8
35–44	3059	110	3.6	2888	199	6.9
45–54	1668	137	8.2	1765	199	11.3
55–64	1020	80	7.8	1064	148	13.9
65^+^	942	94	10.0	935	95	10.2
Total	19 288	464	2.4	19 968	733	3.7

### Sociodemographic characteristics of people with podoconiosis in the phase 2 study

A total of 366 people with podoconiosis were approached for the detailed study in four randomly selected ‘kebeles’. These participants consisted of 134 (36.6%) males and 232 (63.4%) females. On average, the respondents had lived in the study area for 38.64±18.24 years. The majority of the respondents (337 (92%)) belonged to the economically active age group. More than 75% had no formal education, and the majority (346, 94.5%) of the study participants were farmers (table 3).

**Table 3:** Sociodemographic characteristics of people with podoconiosis in the phase 2 study in Wayu Tuka ‘woreda’, January to March 2015 (n=366)

Characteristics	Category	N (%)
Sex	Male	134 (36.6)
	Female	232 (63.4)
Age	15–24	25 (6.8)
	25–34	54 (14.8)
	35–44	88 (24)
	45–54	87 (23.7)
	55–64	67 (18.3)
	65^+^	45 (12.3)
Educational status	Cannot read or write	279 (76.2)
	Can read and write	87 (23.8)
Occupation	Farming	346 (94.5)
	Others^1^*	20 (5.5)
Marital status	Single	81 (22.1)
	Married	208 (56.8)
	Others^2^*	77 (21.0)

*Others^1^, begging, students, retired; Others^2^, widowed, divorced, separated.

### Clinical characterisation of podoconiosis in the phase 2 study

People with podoconiosis in the four randomly selected ‘kebeles’ were categorised into five clinical stages according to pre-existing clinical staging criteria.^17^ The majority (159, 43.4%) had clinical stage 2 disease. The greatest below-knee leg circumference was 26.6±5.3 cm (range 18–45). Table 4 summarises clinical characteristics of podoconiosis.

**Table 4:** Clinical characterisation of people with podoconiosis in the phase 2 study in Wayu Tuka ‘woreda’, January to March 2015

Characteristics	Category	N (%)
Clinical stage (n=366)	One	2 (0.5)
	Two	159 (43.4)
	Three	115 (31.4)
	Four	66 (18.0)
	Five	24 (6.6)
Mossy appearance (n=366)	Yes	141 (38.5)
	No	225 (61.5)
Wound (n=366)	Yes	138 (37.7)
	No	228 (62.3)
Leg circumference (cm, n=359)	18–27	220 (61.3)
	28–36	123 (34.3)
	37–45	16 (4.5)

### Features of ALA

ALA is the major cause of morbidity in people with podoconiosis. The majority (325, 88.8%) of people with podoconiosis had experienced ALA in the year before the survey. The most recent episode of ALA had occurred an average of 2.97 weeks before the study. On average, people with podoconiosis experienced 23.3±14.4 ALA episodes/year and, on average, were bed bound for 6.42±5.39 days, meaning that, on average, each person with podoconiosis lost 149.5 days of activity per year. Nearly half (182, 49.8%) of the people with podoconiosis had ALA at the time the interview was conducted. More than one-third (38.4%) of people with podoconiosis reported that ALA episodes were worse during the rainy season. The major trigger of an ALA episode was prolonged walking (reported by 163, 44.1%). Many (137, 42.2%) had retired to bed without treatment at least once in the past year as a coping mechanism. During episodes of ALA, 40% of people with podoconiosis had sought treatment at a podoconiosis treatment clinic while 9.5% had not sought treatment of any kind (table 5).

**Table 5:** Features of ALA among people with podoconiosis in Wayu Tuka ‘woreda’, January–March 2015

Characteristics	Category	N (%)
ALA in year before survey (n=366)	Yes	325 (88.8)
	No	41 (11.2)
ALA at time of interview (n=366)	Yes	182 (49.8)
	No	184 (50.2)
Time when ALA worsens (n=325)	Rainy season	142 (38.4)
	All the time	74 (32.1)
	During ‘Chagino’	68 (18.4)
	Hot and dry season	41 (11.1)
Triggers of acute attack (n=325)	Long walk	163 (44.1)
	Hard physical work	66 (17.8)
	Walking barefoot	64 (17.3)
	More work than usual	32 (8.6)
ALA coping mechanisms (n=325)	Stay in bed	137 (42.2)
	Resort to less physical work	120 (36.9)
	Get treatment	68 (20.9)
Place treated (n=366)	Podoconiosis treatment centre	148 (40.4)
	Health facilities*	93 (25.4)
	Traditional	90 (24.5)
	Treatment not sought	35 (9.5)

*Health facilities=health centre, health post, hospital and pharmacy/drug store.

### Factors associated with ALA

In multivariate analysis, never walking barefoot and daily foot washing were associated with lower odds of ALA. People with podoconiosis who never walked barefoot had one-quarter the odds of ALA as those who walked barefoot at times (AOR=0.23, 95% CI 0.06 to 0.80, p=0.025). People with podoconiosis who washed their feet daily had one-twelfth the odds of ALA as those who did not (AOR=0.09, 95% CI 0.01 to 0.75, p=0.023). Variables such as patient's age, clinical stage of disease, mossy appearance and presence of wound were associated in the bivariate analysis, but these associations did not persist after multiple logistic regressions (table 6).

**Table 6:** Factors associated with ALA in people with podoconiosis in Wayu Tuka ‘woreda’, January–March 2015

Variable	ALA		COR (95% CI)	AOR (95% CI)	p Value
	Yes (%)	No (%)			
*Situations in which people with podoconiosis walk barefoot*					
Farming					
Y^*^	83 (22.7)	242 (66)	1.14 (0.54 to 2.29)	1.46 (0.6 to 3.6)	0.403
N^*^	17 (4.6)	24 (6.5)	1	1	
Non-Farming					
Y	74 (20)	251 (68.5)	0.56 (0.24 to 1.3)	0.39 (0.14 to 1.05)	0.063
N	34 (9.2)	20 (5.4)	1	1	
Home					
Y	78 (21.3)	247 (67.5)	1.84 (0.86 to 3.94)	1.48 (0.49 to 4.1)	0.384
N	20 (5.5)	21 (5.7)	1	1	1
Always barefooted					
Y	26 (7.01)	299 (81.7)	0.49 (0.09 to 2.49)	0.41 (0.06 to 2.54)	0.335
N	2 (0.54)	39 (10.6)	1	1	1
Never walk barefooted					
N	204 (55.7)	38 (10.4)	1	1	1
Y	118 (32.2)	6 (1.6)	0.31 (0.13 to 0.71)	0.23 (0.06 to 0.80)	0.025
Wash feet daily					
N	1 (0.27)	42 (11.5)	1	1	1
Y	52 (14.2)	271 (74.2)	0.11 (0.01 to 0.85)	0.09 (0.01 to 0.75)	0.026
Age of patients					
15–24	58 (15.84)	19 (5.1)	1	1	1
25–34	80 (22.13)	8 2.18)	0.40 (0.06 to 2.52)	47.7 (3.96 to 574)	0.06
35–44	81 (22.13)	6 (1.63)	0.20 (0.03 to 1.26)	5.36 (0.63 to 45)	0.124
45–54	61 (16.67)	6 (1.64)	0.15 (0.02 to 0.98)	1.93 (0.30 to 12)	0.482
55–64	43 (11.75)	2 (0.5)	0.19 (0.03 to 1.30)	0.93 (0.14 to 6.19)	0.940
65+	1 (0.27)	1 (0.27)	0.09 (0.01 to 0.84)	1.36 (0.20 to 8.97)	0.744
Clinical stage					
Stage 1	1 (0.27)	1 (0.27)	1	1	1
Stage 2	133 (36.3)	26 (7.01)	0.39 (0.06 to 2.24)	0.56 (0.01 to 22.3)	0.762
Stage 3	102 (27.9)	13 (3.55)	0.26 (0.04 to 1.53)	0.68 (0.01 to 27.9)	0.839
Stage 4	62 (16.9)	4 (1.36)	0.13 (0.01 to 0.93)	0.46 (0.01 to 21.9)	0.697
Stage 5	24 (6.5)	0	–	–	–
Mossy appearance					
Y	194 (53)	32 (8.7)	2.06 (1 to 4.2)	1.19 (0.43 to 3.2)	0.739
N	124 (33.8)	13 (3.5)	1	1	1
Presence of wound					
Y	199 (54.3)	30 (8.2)	1.53 (0.7 to 3.04)	0.55 (0.27 to 2.0)	0.737
N	260 (71.0)	22 (6)	1	1	1

AOR, adjusted OR; COR, crude OR; N*=No; Y**=Yes.

### Social stigma

Approximately one in three (125, 33.8%) people with podoconiosis mentioned that they had experienced one or more forms of social stigma at school, church, within marriage, at the market place or at feasts. Stigma took the form of being excluded, shunned, pointed or gestured at and not having products bought at market.

### Factors related to footwear, personal hygiene and walking experience

The experience and attitudes of respondents towards footwear and personal hygiene were assessed. The majority (348, 94.1%) of people with podoconiosis had no problem finding enough water and took an average of 22 min to reach a water source. The majority (315, 85.1%) of people with podoconiosis washed their feet at least once per day (mean 1.4±0.086), and 79 (21.4%) washed their feet with soap daily. Almost all (345, 94.3%) had washed their feet on the night before the interview was conducted. Foot washing behaviour did not change in 97 (26.2%) people with podoconiosis after their leg started to swell. The mean age at first shoes wearing was 25.94±13.83 (range 4–95) years. During the interview, 162 (43.8%) were wearing closed plastic shoes, while 32 (8.6%) were barefoot. A small but important subset (21, 5.7%) had never worn shoes.

## Discussion

Burden assessment using a household census enables the number of people with podoconiosis within a district to be determined, and provides information on their distribution within the district. Such information is important for planning and implementing podoconiosis interventions in endemic districts. Our study identified that the prevalence of podoconiosis in Wayu Tuka ‘woreda’ in 2015 was 3.05%. The prevalence was higher among females than males and increased with age. The male to female ratio was 1:1.58 and the economically active age group (15–64 years of age) was heavily affected. ALA episodes were a major cause of morbidity, and patients' ability to work was impaired by ALA episodes. Patients also experienced significant stigma in a range of settings. The habits of never walking barefoot and daily foot hygiene were both independently associated with reduced odds of ALA.

At 3.05% (95% CI 2.9% to 3.2%), the prevalence of podoconiosis in those aged 15 years and older in Wayu Tuka ‘woreda’ was slightly more than that reported from Gulliso ‘woreda’ in west Wollega, 2.8%,^5^ but lower than that reported from Debre Elias ‘woreda’ (3.3%) and Dembecha ‘woreda’ (3.4%),^6^ among residents of Midakegn district, central Ethiopia (7.4%),^18^ and Ocholo village (5.1%)^19^ and Wolayta zone (5.5%) of southern Ethiopia.^9^ However, it falls within the earliest national estimate of 0.4% to 3.7% across 56 markets.^20^ Podoconiosis distribution shows significant geographic variation according to level of elevation and precipitation,^13^ ^21^ and the prevalence estimated in Wayu Tuka ‘woreda’ falls within the variation demonstrated in Oromia region in the recent nationwide mapping.^13^ ^21^

Male to female sex ratio was 1:1.58, which is consistent with several recent studies conducted in other areas, including the national study,^22^ Gulliso (1:1.26)^5^ and Gojjam, northern Ethiopia (0.98:1).^6^ The risk in females may be enhanced as they shoulder the greatest burden of agricultural work and are less likely to own shoes than men. In Ethiopia, activities such as fetching water, cooking food, washing clothes, farming and buying goods from local markets are their daily duties and responsibilities. Podoconiosis heavily affected the economically active age group in this study. Studies in northern Ethiopia and Gulliso, west Wollega, have also shown this age group to be strongly affected.^5^

Episodes of ALA had been experienced in the past year by 88.8% of patients, a slightly lower proportion than that found in Gulliso ‘woreda’ (97%). Some patients linked ALA with ‘chagino’ (the time around the new moon, often associated in local understanding with weather changes). ALA was not significantly associated with sex or age at onset of illness, consistent with the study in west Wollega.^5^ Activities were impaired by lymphoedema and ALA in about three-quarters of patients. During ALA, patients experienced severe pain and had to stay in bed for several days. A study conducted in northern Ethiopia showed that three in five patients claimed their ‘movement’ was impaired by podoconiosis and one in four by ALA.^16^ In this study, ALA had considerably more impact on ability to work than previous studies. An average total of 150 days (5 months) was lost by patients in Wayu Tuka ‘woreda’ annually, six times that recorded in Gulliso ‘woreda’ (24 working days lost on average).^5^ Earlier studies showed that people with podoconiosis were about half as productive as unaffected individuals, and that medical costs and productivity losses exceeded US$16 million per year in one zone (with a population of 1.8 million) in southern Ethiopia. According to this study, most patients did not completely stop work, but spent only 3.56±2.87 hours per day on economic activity compared to the 6.52±2.53 hours worked by controls. Female patients spent 3.45 hours less on domestic activity compared to matched controls.^10^

One in four patients (25%) experienced stigma in this study. In contrast, a study conducted in northern Ethiopia showed that about 13% of patients had experienced one or more forms of social stigma at school, church or in the market place.^16^ Stigma is increasingly being recognised as an obstacle to good health and a barrier to accessing healthcare. Health-related stigma affects the life chances of individuals, increasing their exposure to risks and limiting access to protective factors, potentially adding to their burden of disease or disability.^10^ ^16^ The main reasons for the prevailing discrimination against patients and affected families are the erroneous beliefs that podoconiosis cannot be prevented, treated or controlled; that it is associated with curses; and that it runs in families through hereditary factors that make disease inevitable.^23^ ^24^

Most patients could reach a water source in an average 22 min walking round trip. Another study in western Ethiopia also claimed that patients could access water with a walk of ‘only’ 20 min;^5^ however, the WHO defines access to drinking water as ‘availability of at least 20 L/person/day/within 30 min' walk’.^25^ Patients may not have to walk far for water, but whether they can access adequate water is another question. Patients who wash their feet at least once per day are less likely to experience ALA. In Wayu Tuka, 85% of patients washed their feet at least once per day, a larger proportion than in Gulliso (66.7%^14^). However, a lower proportion of patients used soap daily to wash their feet than in Gulliso (21.4% vs 58%, respectively). Using shoes regularly and washing feet with soap consistently were uncommon among study subjects in Wayu Tuka ‘woreda’. Our study showed that there were several challenges to primary and secondary prevention of podoconiosis. Inconsistent shoe-wearing was identified among patients who owned shoes. Although few respondents were barefoot, the shoes they were wearing (open plastic or open tyre) were not protecting them from irritant soil. The other issue was the average age at which they started wearing shoes: at 25 years, this indicated prolonged contact with soil and delay in protection.

This study used a large scale community-based house-to-house survey in all ‘kebeles’ of Wayu Tuka ‘woreda’, followed by a detailed study of patients with podoconiosis. This provides a complete profile of people with podoconiosis in the district. Nonetheless, the study is not without limitations. A clinical algorithm was used to differentiate podoconiosis from other disease causing leg swelling in such areas. Had a serological test been available, it would have helped exclude lymphatic filariasis, the most common phenocopy in tropical areas. However, given that the study area is at 2200 m above sea level, transmission of filaria is extremely unlikely.

We recommend strong prevention and morbidity management interventions to address this developmental challenge. These interventions will require collaboration between local government and non-government stakeholders, and integration with existing programmes addressing water and sanitation, neglected tropical diseases and chronic diseases.
