# Supplementary material for: Burden assessment of podoconiosis in Wayu Tuka woreda, east Wollega zone, western Ethiopia: a community-based cross-sectional study
Source: BMJ Open. 2016 Sep 23;6(9):e012308. doi: 10.1136/bmjopen-2016-012308 (PMC5051403; doi:10.1136/bmjopen-2016-012308)
Supplement: supplementary file [file bmjopen-2016-012308supp_file2.pdf]

**Supplementary File 2**

Interviewer's name (HEW) ________________________HEW code_______sign.____

Date_____/____/____

**Registration Format (Wayu Tuka Woreda)** Kebele____________ 'Zone'_________

| No | HH | HH mem | Do you know any patient With podoconiosis in your family? | How many? | First name | Fathers name | Sex 1.M 2.F | Age | Marital status |
|---|---|---|---|---|---|---|---|---|---|
| # | # | # | 1 Yes2.No | # | | | | # | # |
| 1 | | | | | | | | | |
| 2 | | | | | | | | | |
| 3 | | | | | | | | | |
| 4 | | | | | | | | | |
| 5 | | | | | | | | | |
| 6 | | | | | | | | | |
| 7 | | | | | | | | | |
| 8 | | | | | | | | | |
| 9 | | | | | | | | | |
| 10 | | | | | | | | | |

**Detailed study**

**Kebele____________________Zone______Nurse______________________Nurse code_____**

***Consent:*** *we would like to ask you some questions to understand the problem of podoconiosis in your area. We also want to examine your legs .we hope that this will help plan services for people with this condition. Thank you for participating in this study.*

**1. REGISTRATION**

1.1. Name________________________________ 1.2. Sex: 1.Male 2. Female 1.3.Age _____years

1.4 .Number of years lived in this kebele_____________ years

**2. HISTORY OF DISEASE AND PERCEPTION ABOUT PODOCONOISIS**

2.1 How old were you (age) when you first noticed your legs are swollen? _______ Years

2.2 How long after the swelling you try to get treatment care? ________ Years

2.3 Where did you go first?

1. Health center
2. Health post
3. Hospital
4. Podoconiosis treatment center
5. Pharmacy/drug store
6. Traditional/healers
7. Others ____________________

2.4 When were you last sick with a painful swollen leg?

1. ____wks ago
2. _____months ago
3. ._____years ago
4. Never (Go to Q 2.6)

INTERVIEWER: MAY I LOOK AT YOUR LEG?

2.5.1 Has the person got acute attack during the interview?

1. Yes
2. No (GO TO Q2.6.1)

2.5.2. If yes to Q2.5.1: what signs and symptoms do you observe?

1. Oozing
2. Tenderness (Pain in touch)
3. 3. Hot (on touch)
4. 4. Fever
5. 5. Swollen lymph node

2.6.1 Did you look for help?

1. Yes
2. No (GO TO Q2.6.3)

2.6.2. If 'YES' to Q2.6.1: where did you seek help?

1. Health center
2. Health post
3. Hospital
4. Podoconiosis treatment center
5. Pharmacy/ drug store
6. Traditional healers

2.6.3 Did you stay in bed?

1. Yes
1. No (Go to Q2.6.5)

2.6.4 If "Yes" to Q2.6.3: How long you stay in bed? _______days

2.6.5. How often in the last 12 months did you have this problem? ___times /every___months

2.6.6. Which time of the year is this problem worse?

1. All the time
2. Rainy season
3. Hot and dry season
4. During chagino

5. Others ____

2.6.7. What precipitate acute attack symptoms?

1. Hard (labours) work
2. A little more than usual work
3. Long walk
4. Other ______

2.6.8. How do you usually cope with (react towards) acute attack episodes?

1. Resort to less exertive work
2. Stay in bed
3. Other (specify) ___________

**PERCEPTION ON CAUSE, PREVENTION AND CONTROL OF PODOCONIOSIS**

2.7. Why do you think you have podoconiosis? (NEVER READ ANSWERS.PROBE. CIRCLE ALL THAT APPLY)

1. I am barefooted
2. I or my families are cursed
3. This is a familial problem (it runs through 'blood/ bone' of our family)
4. I got it following contagion with an affected person (e.g. wore his/her shoes)
5. Following snake bite
6. Following exposure to condensation
7. I don't know

2.9. Do you think podoconiosis can be prevented?

1. Yes
2. No - CIRCLE AND GO TO Q2.11
3. I don't know( GO TO Q 2.11)

2.10 If the answer to Q2.9 was ‘Yes’: How can podoconiosis be prevented? (PLEASE DON’T READ THE ANSWERS. PROBE AND CIRCLE ALL THAT APPLY)

1. Wearing shoes protective of exposure to soil regularly
2. Washing feet with soap and water regularly
3. Avoiding marriage with affected families
4. Avoiding contact with affected

2.11. Do you think progression of podoconiosis can be controlled at early stages of the condition/is it curable?

1. Yes
2. No …CIRCLE AND GO TO Q2.13
3. I don’t know …. GO TO Q2.13

2.12. If the answer to Q 2.11 was ‘Yes’ how can podoconiosis be controlled at early stage? (PLEASE DON’T READ THE ANSWERS PROBE AND CIRCLE ALL THAT APPLY)

1. Wearing shoes protective of exposure to soil regularly
2. Washing feet with soap and water regularly
3. Avoiding marriage with affected families
4. Avoiding contact with affected

2.13. If the answer to Q2.12 was No: why do you think podoconiosis cannot be controlled (cured)? (PLEASE DON’T READ THE ANSWERS PROBE AND CIRCLE ALL THAT APPLY)

1. It is familial (runs through ‘blood/bone’)
2. There is no drug for it
3. I have never seen a cured person
4. Others specify_____________

**SOCIAL STIGMA**

**2**.8. Did you experience any social stigma / discrimination because you have podoconiosis? (READ B-F ONE BY ONE. IF THEY ANSWER YES, UNDERLINE THE POINTS MENTIONED IN THE BRACKET. PROBE FOR OTHER EXPERIENCES PUT THAT UNDER 7)

1. No .people treat me like any person else.
2. Yes, schooling (drop out, shunning pointing fingers, pinching nose)
3. Yes, church (exclusion, shunning, pointing fingers, pinching nose)
4. Yes, marriage (exclusion, shunning, pointing fingers, pinching nose)
5. Yes, market place (people are not interested to buy my products)

6. Yes, feasts (exclusion, shunning, pointing fingers, pinching nose)
7. Others (specify) ____________________

**2.14 Examination**

| | 2.14.1 stage 1-5 | 2.14.2 mossy 1. Yes 2. No | 2.14.3 wound 1. Yes 2. No | 2.14.4 type( watery, bag, nodular, mixed) | 2.14.5Greatest below knee leg circumference (cm) |
|---|---|---|---|---|---|
| 1. right leg | | | | | |
| 2. left leg | | | | | |

**3. BACKGROUND**

3.11. Educational status:

1. cannot read and write
2. Read and write

3.12. If the answer to Q 3.11 is (2) what grade are you? _____

3.2 Main occupation

1. Farming
2. Weaving
3. Daily labourer
4. House
5. Others___________

3.3. Marital status

1. Single.
2. Married
3. Widowed
4. Divorced
5. Separated

**3.4. FAMILY HISTORY**

(NOTE: THIS PART IS A VERY SENSITIVE, YET RELEVANT SECTION. SO YOU SHOULD BE EXTRA POLITE AND EXPLAIN THAT ALL INFORMATION WILL BE KEPT CONFIDENTIAL. IN ADDITION MENTION THAT THIS INFORMATION WILL BE RELEVANT FOR TARGETING DISEASE PREVENTION AND TREATMENT PROGRAMS)

3.4.1. Does any member of your family (meaning relatives such as parents, grandparents, cousins, siblings, or children) have podoconiosis?

1. Yes
2. No_______ GO TO Q3.4.3

3.4.2 If "Yes" to Q3.4.1: how are you related? (PROBE FOR OTHER CATEGORIES)

| | a. # | b. sex 1.M 2.F | Still alive? 1. Y2. N |
|---|---|---|---|
| 3.4.2.1 Grandparent? | | | |
| 3.4.2.2parent? | | | |
| 3.4.2.3sibling? | | | |
| 3.4.2.4child? | | | |
| 3.4.2.5other? | | | |

3.4.3 How many member of your household (together with you) are affected? _______

3.4.4 Is that person available for interview? 1. Yes 2. No

NOTE TO INTERVIEWER: IF THE ANSWER IS "YES", RECORD THE FOLLOWING AND GIVE APPOINTMENT FOR NEXT DAY INTERVIEW BY A NURSE.

Name _____________________ Age____ Sex 1. Male 2. Female

3.4.5.1 Are any children in the house complaining of problems with feet? 1. Yes 2. No

3.4.5.2 If Yes to Q3.4.5.2: what are the signs and symptoms?

_______________________________________________________________

NOTE TO INTERVIEWER: IF THE ANSWER IS "YES", RECORD THE FOLLOWING AND GIVE APPOINTMENT FOR NEXT DAY INTERVIEW BY A NURSE.

Name _____________________ Age____ Sex 1. Male 2. Female

3.4.6 Why do you think there are multiple affected family members in your Family (household)? (CIRCLE ALL ANSWERS SPECIFIED)

1. Because the disease is contagious
2. Because the disease runs through families (blood/ bone)
3. Because the family is cursed/ any spiritual connection
4. Because the family is poor and cannot afford shoes
5. I don't know
6. Others specify
7.

**4. WEARING SHOES**

4.1.1. How old were you when you first got shoes? ____ Years

4.1.2 How old were you when you first got shoes? ____ Years

4.2 interviewers: observe and describe the shoes the person is now wearing.

1. Hard plastic open
2. Hard plastic covered
3. ‘Barabaso’
4. Leather
5. ’Shara’/canvass
6. Bare footed

4.3. When you usually not wear shoes?

1. During farming
2. During Non –Farming Work
3. At Home
4. I Am Usually Bare Footed
5. I Am Always Barefooted

4.4. How many pairs of shoes do you have? __________

4.5. How many pairs of shoes do you need / year? __________

**5. WASHING FEET**

5.1 can you get enough water?

1. Yes (go to q 5.3)
2. No

5.2 If no q5.1: what was the problem?

1. Seasonal shortage
2. Distance
3. Others specify_____

5.3. How long (in minutes) do you go to the nearest water source (1 way)? _____ Minutes

5.4. Did you wash your feet last night?

1. Yes
2. No

5.5 If yes to q5.4. Interviewer: please observe and describe the cleanliness of the feet

1. Clean and intact
2. Dirty
3. Cracked
4. Dirty and cracked

5.6 How many times do you wash your feet? _____ Times

5.7. After your feet started swelling, did your foot washing behaviours changed?

1. Yes
2. No (go to q 5.9)

5.8. If yes to q5.7: do you wash your feet more often / less often?

1. More often
2. Less often

5.9. Do you use soap for your feet?

1. Yes
2. No

5.10. If "yes" to q5.6: how many times/ times do you wash your feet with soap? _______times

**DISTANCE WALKED**

6.1. How long does it take to go to your nearest field? ________min/hrs

6.2. How many times do you go to your field each month? _________times

6.3. How long does it take to go to your nearest market month? _______minutes

6.4. How often do you go to market each month? ______times

6.5. ASK ANOTHER PERSON OF THE HOUSEHOLD: How many times last year was the person sick with acute attack? _______________________

**7. DISABLING EFFECTS (FUNCTIONAL IMPAIRMENT)**

7.1. Over the past 30 days, has podoconiosis or acute attack of ALA hampered movement (walking, travelling to markets etc) (MORE ONE ANSWER POSSIBLE)

1. Yes, effect of the disease
2. Yes effect of acute attacks
3. Uncertain
4. No

7.2. ASK WOMEN: over the past 30 days, has podoconiosis or acute attack of ALA hampered any house hold chores (cleaning, cooking, child care etc) (MORE THAN ONE ANSWERS POSSIBLE)

1. Yes, effect of the disease
2. Yes effect of acute attacks
3. Uncertain
4. Not applicable (circle this if the respondent is a man)

7.3. ASK MEN: Over the past 30 days, has podoconiosis or acute attack of ALA hampered daily/ occupational work? (MORE THAN ONE ANSWER IS POSSIBLE)

1. Yes, effect of the disease

2. Yes effect of acute attacks
3. Uncertain 4.not applicable (if a woman)

7.4. If either 7.2 or 7.3 is 'yes': what was the effect of the disease? (Read each answer & circle all that apply)

1. Work less hours
2. Worked less energy
3. Absence from work
4. Earned less income
5. Reduced productivity (e.g. harvest from agriculture)
6. Others (specify) __________

7.5. How has podoconiosis affected your work life over a long period of time (many months or years)? (Read each answer and circle all that apply)

1. I work only occasional
2. I work less hours
3. I restored to less remunerative work
4. I avoided physically demanding tasks
5. I totally stopped work
6. Other __________________

7.6. How do you rate your financial status compared to your fellow neighbours with the same occupation and family size?

1. About the same
2. I am poorer
3. I am better off

7.7. If the answer to q 7.6 was '2': did your financial status worsen after your family became affected by podoconiosis?

1. Yes
2. No

**We have finished the interview. Thank you for time and participation!**

**Appendix 2: Standard Training Material Used To Train Nurses and HEWs**

**Podoconiosis: Differential Diagnosis, & Clinical Staging (Professor G. Davey)**

Differential diagnosis - which other conditions could it be?

1• Filarial Lymphoedema 3• Leprosy

2• Onchocerciasis 4• deep fungal infection

**1. Filarial lymphoedema**

**Ask**: Is patient from lowland area? Did the swelling start at the foot or higher up?

**Look**: Is leg swelling very asymmetrical (one leg 'normal')?

**Examine**: Check the groin for swollen lymph node

**Do**: Refer for tablet treatment at Health Centre, invite back for usual MFTPA leg care.

**2.Onchocerciasis**

**Ask**: Does patient have itchy trunk or nodules?

**Look**: Look for onchodermatitis, pretibial depigmentation, onchocercomas, eye changes

**Examin**e: Examine skin of whole body, check the groin for lymph nodes

**Do**: Refer for treatment of onchocerciasis, invite back for usual MFTPA leg care.

**3. Leprosy**

- Exaggerated eyebrow area
- Shiny skin on feet
- Toes may look short
- Foot ulcer may be present
- Patient cannot feel

**Ask:** Has the patient noticed any skin changes?

**Look:** Are there rough raised skin lesions?

**Examine:** For thickened nerves at the elbow or behind the ear; for foot ulcers; check if patient can feel you touch his feet?

**Do**: Refer for leprosy treatment. Invite back for follow up MFTPA foot care.

**5. Fungal**

Deep fungal infection Lump on one foot May have small black dots on surface

**Do**: Refer to surgeon for biopsy, anti-fungal treatment/amputation

**CLINICAL STAGING**

**Why is staging important?**

- So program planners can assess burden of disease in given area;

- So patients can see effect of self-treatment;
- So health professionals can document effectiveness of medical and surgical treatment;
- So researchers can document effects of public health interventions.

**Stage 0.** No disease present.

**Stage 1**.Swelling reversible overnight.

**Stage 2**.Persistent below-knee swelling; if present, knobs or bumps are below the ankle ONLY

**Stage 3**. Persistent below-knee swelling; knobs or bumps present above the ankle.

**Stage 4** .Persistent above-knee swelling; knobs or bumps present at any location.

**Stage 5**. Joint fixation; swelling at any place in the foot or leg. (X-rays may show tuft erosion and loss of bone density)
